# Modeling the Effects of Multiple Intervention Strategies on Controlling Foot-and-Mouth Disease

**DOI:** 10.1155/2015/584234

**Published:** 2015-10-04

**Authors:** Steady Mushayabasa, Gift Tapedzesa

**Affiliations:** Department of Mathematics, University of Zimbabwe, P.O. Box MP 167, Harare, Zimbabwe

## Abstract

Foot-and-mouth disease (FMD) is a threat to economic security and infrastructure as well as animal health, in both developed and developing countries. We propose and analyze an optimal control problem where the control system is a mathematical model for FMD that incorporates vaccination and culling of infectious animals. The control functions represent the fraction of animals that are vaccinated during an outbreak, infectious symptomatic animals that are detected and culled, and infectious nonsymptomatic animals that are detected and culled. Our aim was to study how these control measures should be implemented for a certain time period, in order to reduce or eliminate FMD in the community, while minimizing the interventions implementation costs. A cost-effectiveness analysis is carried out, to compare the application of each one of the control measures, separately or in combination.

## 1. Introduction

Foot-and-mouth disease (FMD) is a highly contagious viral disease of cloven-hoofed animals and is one of the most economically important diseases of livestock [1]. The causative agent of the disease is a small icosahedral nonenveloped RNA virus classified within the* Aphthovirus* genus, as a member of the Picornaviridae [2]. The virus is airborne and can also be transmitted through physical contact with infected animals' expired air, saliva, milk, urine, semen, animal feed and bedding, and so forth [3]. Direct or indirect contact with FMD-infected animals can result in susceptible animals becoming diseased or subclinically infected [2]. The incubation period for FMD can vary with the species of animal, the dose of virus, the viral strain, and the route of inoculation [4]. It is reported to be 1 to 12 days in sheep, with most infections appearing in 2–8 days, and 2 to 14 days in cattle [5]. After the incubation, a fraction of infected animals progress to symptomatic stages and the remainder become FMD carriers. FMD carriers are defined as animals in which either viral nucleic acids or live virus can be found for more than 28 days after infection [4]. How long an animal can remain a carrier varies with the species [6]. Most cattle carry foot-and-mouth disease virus (FMDV) for six months or less, but some animals can remain persistently infected for up to 3.5 years [4, 5]. The virus or its nucleic acids have been found for up to 12 months in sheep, up to 4 months in goats, for a year in water buffalo, and up to 8 months in yaks [7]. Carrier animals can only be identified by collecting esophageal-pharyngeal fluids for virus isolation and/or the detection of nucleic acids. Repeated sampling may be necessary to identify a carrier, as the amount of virus is often low and fluctuates [4].

Conventional control measures of the disease include movement restriction—for example, through construction of “veterinary boundaries”—that is, cordon fences erected to divide a country into multiple subregions to restrict the movement of animals across the borders; educational awareness; quarantine; vaccination and culling of detected infected animals [3]. Practising import regulations can be essential to prevent FMDV from being introduced from endemic regions in infected animals or contaminated foodstuffs fed to animals [4].

Since the 2001 FMD outbreak in the United Kingdom mathematical modeling of FMD has been an interesting topic for a number of researchers; see, for example, [3, 8–11]. Tildesley et al. [8] proposed a probabilistic FMD transmission model to explore an optimal deployment strategy of limited reactive ring vaccination of cattle in a single epidemic outbreak. Their work suggested that optimal ring size is highly dependent upon logistic constraints. In 2011, Hansen and Day [10] constructed an SIR, Susceptible-Infectious-Removed, model to assess the impact of limited isolation resources and limited vaccination resources. Results from their study highlighted a number of areas that warrant further study and also emphasized the impact of time-optimal control on controlling the generation of new infections in resource-limited settings. More recently, Ringa and Bauch [3] developed an SEIRVC, Susceptible-Exposed-Infectious-Removed-Vaccinated-Culled, pair-approximation model of FMD transmission in a near-endemic population. Their work suggested that the optimal long-term control of FMD by vaccination in near-endemic settings can be achieved by rolling out a prophylactic vaccine as much as possible, especially if resources are limited.

The primary goal of this paper is to formulate a model for FMD dynamics that includes relevant biological detail and accounts for multiple intervention strategies. The FMD intervention strategies to be considered are pre- and postexposure vaccines and culling of symptomatic and nonsymptomatic infectious animals. During an FMD outbreak, a vaccine may be protective or suppressive. Suppressive vaccination reduces the potential of FMDV production in herds and flocks that may already have been exposed to infection [12]. Vaccination of exposed animals often arose due to the fact that it is difficult to detect an animal in this stage of disease as the acute phase of virus replication may be transient [12, 13]. By vaccinating all the exposed animals, it is hoped that those not already infected will develop sufficient immunity to provide at least partial protection against clinical disease [12]. Protective vaccination is used on herds and flocks that are in the vicinity of an outbreak but are thought not to have been exposed [12]. Prior studies suggest that a vaccinated exposed animal is highly likely to progress to be an FMD carrier [14]. Apart from suppressive and protective vaccines, our model explores the role of culling infectious symptomatic and nonsymptomatic animals.

## 2. Methods and Results

### 2.1. Model Framework

The total population of livestock is subdivided into proportions of susceptible animals *S*(*t*), vaccinated animals *V*(*t*), latently infected animals not vaccinated *E*(*t*), exposed animals that have been administered a suppressive vaccine *E*_*v*_(*t*), infectious animals displaying clinical signs of the disease *I*(*t*), and infectious animals not displaying clinical signs of the disease *I*_*c*_(*t*), also known as FMD carriers. Thus the total population is

(1) Nt=St+Vt+Et+Evt+It+Ict.

Susceptible animals acquire FMD infection at rate *λ* = *β*[*I* + (1 − *ϵ*)*I*_*c*_], where *β* denotes the FMD transmission parameter, which is considered to be a product of the between-animal contact rate *c*—that is, the average number of contacts between animals per unit of time—and the FMDV transmission probability per each contact *p*; that is, *β* = *cp*. Factor (1 − *ϵ*), accounts for the reduced probability of FMD transmission by animals in class *I*_*c*_. If *ϵ* = 1 it implies that animals in class *I*_*c*_ do not transmit FMDV, while 0 < *ϵ* < 1 implies that animals in class *I*_*c*_ have less chance of infecting susceptible animals. Animals in class *V* acquire FMD infection at rate (1 − *θ*)*λ*, where *θ* captures the impact of vaccination on reducing the susceptibility of animals in this class to FMD infection, that is, the vaccine efficacy. The model takes the following form:

(2) S˙=μ−λS−μ+αS,V˙=αS−1−θλV−μV,E˙=λS−μ+γE,E˙v=1−θλV+fγE−μ+ωEv,I˙=1−κ1−fγE−μ+σdI,I˙c=ωEv+1−fκγE+1−σdI−μ+ξ+δIc,

where the upper dot represents the derivative of the component with respect to time. The constant parameter *μ* denotes birth or natural permanent exit of animals from the community. Susceptible animals acquire protective vaccination at per capita rate *α*, *γ* denotes the incubation period of animals in class *E* and this is usually in the range of 2–14 days [13], and *f* is the proportion of exposed animals in class *E* that receive suppressive vaccination and the remainder (1 − *f*) that do not receive suppressive vaccination progress to infectious carrier *I*_*c*_ or symptomatic infectious animals *I*. It is assumed that a fraction *κ* of nonvaccinated exposed animals progress to the infectious carrier population and the complementary proportion (1 − *κ*) becomes symptomatic and infectious. Further, we assume that vaccinated exposed animals *E*_*v*_ progress only to the infectious carrier population at rate *ω*; *d* accounts for the permanent exit rate of animals in class *I* due to FMD infection, with a fraction (1 − *σ*) progressing to infectious carrier population and the remainder *σ* succumbing to disease-related mortality; *δ* ^−1^ is the average life-span of an infectious carrier animal. Prior studies suggest that in cattle population an FMD carrier may exist for a period of 3.5 years [4]; *ξ* denotes the detection and culling rate of infectious FMD carrier animals.

**Figure 1:**
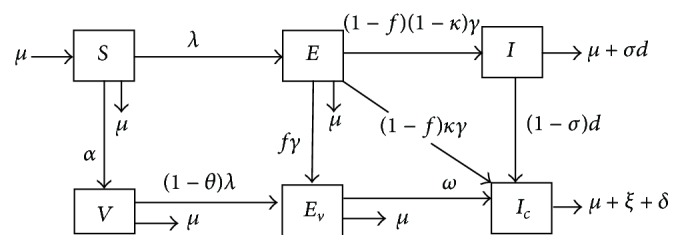
Model structure.

Our assumptions on the transfer of the population are demonstrated in Figure 1.

For biological reasons, we study the solutions of system (2) in the closed set:
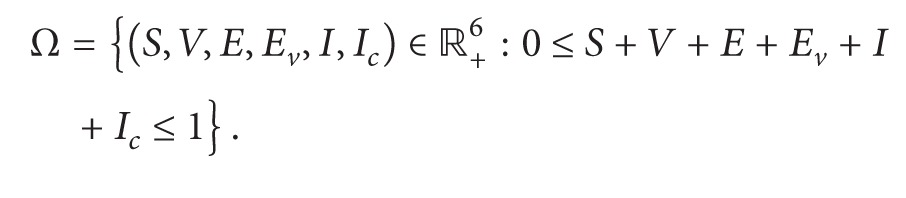
(3)
*Ω* is positively invariant. Thus all solutions of system (2) with nonnegative initial data will remain nonnegative for all time.

### 2.2. Equilibrium Points and Stability Analysis

*Infection-Free Equilibrium Point*. In the absence of FMD in the community, system (2) admits an equilibrium point known as infection-free, and it is given by

(4) E0S0,V0,E0,Ev0,I0,Ic0=μα+μ,αα+μ,0,0,0,0.

Following the next-generation method [16], the reproductive number of system (2) is

(5) RA=β1−ϵ1−θαωα+μμ+ωμ+ξ+δ+β1−ϵfγω+μ+ω1−fκγμα+μμ+γμ+ωμ+ξ+δ+β1−f1−κγ1−ϵ1−σd+μ+ξ+δμα+μμ+γμ+σdμ+ξ+δ.

*ℛ*_*A*_ is a threshold for disease invasion or eradication, under suitable conditions, such as the absence of a backward bifurcation. See [17] for more discussion. Theorems 1 and 2 are based on computations in Appendix A.

**Theorem 1 .**

The FMD-free equilibrium point *ℰ* ^0^ is globally asymptotically stable whenever *ℛ*_*A*_ ≤ 1.

**Theorem 2 .**

The unique endemic equilibrium *ℰ* ^*∗*^ of system (2) is locally asymptotically stable for *ℛ*_*A*_ > 1 but close to one.

*Sensitivity Analysis of the Reproductive Number*. Sensitivity analysis of model parameters is very important to design and control strategies as well as being a direction to future research. Local sensitivity indices allow us to measure the relative change in a state variable when a parameter changes. In computing the sensitivity analysis, we adopt the approach described by Arriola [18]. The normalized forward sensitivity index of a variable to a parameter is the ratio of the relative change in the variable to the relative change in the parameter. When the variable is a differentiable function of the parameter, the sensitivity index may be alternatively defined using partial derivatives.

**Definition 3 .**

The normalized forward sensitivity index of a variable, *u*, that depends differentiably on a parameter, *p*, is defined as

(6) $$\begin{array}{c} \Gamma_{p}^{u}≔\frac{\partial u}{\partial p}\times \frac{p}{u}. \end{array}$$

Model parameters whose sensitivity index values are near −1 or +1 suggest that a change in their magnitude has a significant impact on either increasing or decreasing the size of the reproductive number *ℛ*_*A*_. From Table 2, it is clear that *ℛ*_*A*_ is most sensitive to *β*, *θ*, *ξ*, and *ϵ*. An increase in *β* by 10% would increase *ℛ*_*A*_ by 10%. An increase in *θ* by 10% would decrease *ℛ*_*A*_ 9.9%. Similarly, if *ξ* increases by 10%, then *ℛ*_*A*_ decreases by 9.9%. In summary, the numerical estimation of the indices suggests that reduction in disease-transmission rate coupled by an increase in preexposure vaccine efficacy and culling of infectious FMD carriers can lead to a significant reduction in new FMD cases.

### 2.3. Optimal Control

In this section, we formulate an optimal control problem for the transmission dynamics of FMD by extending the autonomous system (2). The goal here is to study the best strategies to curtail the epidemic. Three intervention methods, called controls, are included in our initial model. Controls are represented as functions of time and assigned reasonable upper and lower bounds. The first control *u*_1_(*t*) attempts to strengthen the impact of vaccination and the second control *u*_2_(*t*) attempts to strengthen the impact of detection and culling of infectious symptomatic infectious animals while the third control *u*_3_(*t*) attempts to strengthen the impact of detection and culling of infectious nonsymptomatic animals. Using the same parameter and class names as in system (2) and Table 1, the system of differential equations describing our model with controls is

(7) S˙=μ−βI+1−ϵIcS−μ+u1αS,V˙=u1αS−β1−θI+1−ϵIcV−μV,E˙=βI+1−ϵIcS−μ+γE,E˙v=β1−θI+1−ϵIcV+u1γE−μ+ωEv,I˙=1−u11−κγE−μ+u2dI,I˙c=ωEv+1−u1κγE+1−u2dI−μ+ξu3+δIc.

The objective functional, *J*, is used to formulate the relevant optimization problem: finding the most effective strategy that reduces or eliminates the levels of FMD at minimal cost. This minimization goal will be achieved through the implementation of controls *u*_1_(*t*), *u*_2_(*t*), and *u*_3_(*t*) over the preselected time interval [0, *T*]. Mathematically, this corresponds to the minimization of the functional *J* over a set of feasible, (*u*_1_(*t*), *u*_2_(*t*), *u*_3_(*t*)), strategies on [0, *T*]. The *J* functional is defined as follows:
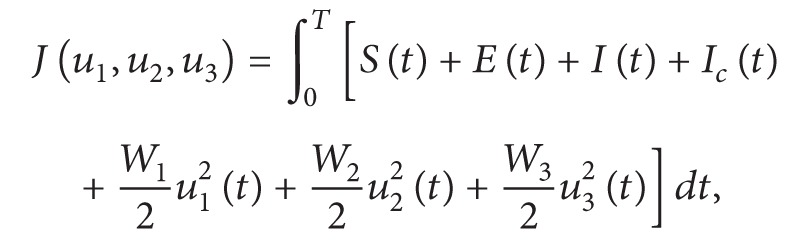
(8)where the constants *W*_*j*_, *j* = 1,2, 3, are a measure of the relative cost of the interventions associated with the controls *u*_1_, *u*_2_, and *u*_3_, respectively.

We consider state system (7) of ordinary differential equations in *ℝ* ^6^ with the set of admissible control functions given by

(9) Γ=u1t,u2t,u3t∈L10,T ∣ 0≤u1t,u2t,u3t≤1,  ∀t∈0,T.

We consider the optimal control problem of determining *S* ^*∗*^(*t*), *V* ^*∗*^(*t*), *E* ^*∗*^(*t*), *E*_*v*_^*∗*^(*t*), *I* ^*∗*^(*t*), and *I*_*c*_^*∗*^(*t*) associated with an admissible control treble (*u*_1_^*∗*^(*t*), *u*_2_^*∗*^(*t*), *u*_3_^*∗*^(*t*)) ∈ Γ on the time interval [0, *T*], satisfying (7), given the initial conditions *S*(0), *V*(0), *E*(0), *E*_*v*_(0), *I*(0), and *I*_*c*_(0) and minimizing the cost functional (8); that is,

(10) Ju1∗t,u2∗t,u3∗t=minΓ⁡ Ju1t,u2t,u3t.

The existence of optimal controls follows from standard results in optimal control theory [19].  Theorem 4 follows from Appendix B.

**Theorem 4 .**

Problem (7)–(10) with given initial conditions *S*(0), *V*(0), *E*(0), *E*_*v*_(0), *I*(0), and *I*_*c*_(0) and fixed final time *T* admits a unique optimal solution (*S* ^*∗*^(*t*), *V* ^*∗*^(*t*), *E* ^*∗*^(*t*), *E*_*v*_^*∗*^(*t*), *I* ^*∗*^(*t*), *I*_*c*_^*∗*^(*t*)) associated to an optimal control treble (*u*_1_^*∗*^, *u*_2_^*∗*^, *u*_3_^*∗*^) on [0, *T*].

The optimal control treble predicted by Theorem 4 represents the optimal intervention strategy, given cost constraints, and can be found by the application of the Pontryagin maximum principle [19].

*Numerical Results*. In this section, we explore the role of optimal control on minimizing cumulative FMD infections in the community. To achieve this, we solve system (7) with a guess for the controls over the time interval [0, *T*] using a forward fourth-order Runge-Kutta scheme and the transversality conditions *λ*_*i*_(*T*) = 0, *i* = 1,2,…, 6. Then, system (B.2) is solved by a backward fourth-order Runge-Kutta scheme using the current iteration solution of system (7). The controls are updated by using a convex combination of the previous controls and the values from (B.3). The iteration is stopped when the values of the unknowns at the previous iteration are very close to the ones at the present iteration.

In all the simulations performed in this section, all parameters are fixed according to Table 1, together with the following estimated initial population levels and controls: *S*(0) = 0.94, *V*(0) = 0, *E*(0) = 0.03, *E*_*v*_(0) = 0, *I*(0) = 0.02, *I*_*c*_(0) = 0.01, *u*_1_ = 0.9, *u*_2_(0) = 0.85, and *u*_3_ = 0.8. For the weights, we assumed that vaccination is the most expensive intervention due to the fact that it involves a large number of animals; hence the cost associated with control *u*_1_(*t*) is assumed to be higher than the cost tied to culling infectious animals, whether symptomatic or nonsymptomatic; thus *W*_1_ > (*W*_2_, *W*_3_). Further, we assumed that detection and culling of infectious nonsymptomatic animals involves a number of procedures such as taking blood samples for examination in laboratory since these animals do not display clinical signs of the disease, compared to detection and culling of symptomatic infectious animals; thus *W*_2_ < *W*_3_. Specifically, we have assigned our weights *W*_1_ = 0.00005, *W*_2_ = 0.00002, and *W*_3_ = 0.000035.

**Figure 2:**
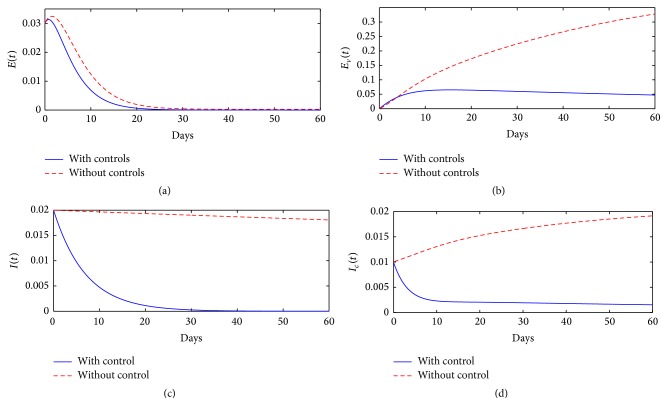
Time-series plots showing the impact of optimal intervention strategies on controlling FMD prevalence in the community, over a period of 60 days.

Figure 2 illustrates the impact of time dependent intervention strategies on controlling FMD prevalence in the community over a period of 60 days. Results from these simulations demonstrate that the presence of optimal intervention strategies can lead to a significant decrease in cumulative FMD cases; the epidemiological classes *E*(*t*) and *I*(*t*) die out after 30 days and 40 days, respectively, of implementing the methods.

**Figure 3:**
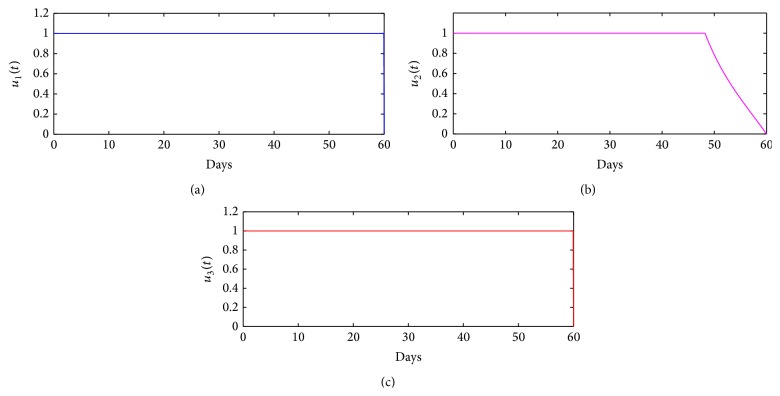
Control profiles for control function: (a) *u*_1_(*t*), (b) *u*_2_(*t*), and (c) *u*_3_(*t*), over a period of 60 days.

The solution for the optimal control problem (7)–(10) is illustrated in Figure 3. From these simulation results, we note that vaccination control, *u*_1_, and culling of infectious nonsymptomatic animals control, *u*_3_, must be maximum for the entire period of 60 days during which the interventions last, while the detection and culling of infectious symptomatic animals control, *u*_2_, should stay at its maximum intensity, for approximately 50 days. The total relative cost for all the strategies is estimated by computing the approximate area under control functions with daily costs computed by dividing the total costs by the number of days where nonzero controls are applied [20]. In this case, the total relative cost for the 60-day strategy—*W*_1_ · (∫_0_^*T*^ *u*_1_(*t*)*dt* = 60), *W*_2_ · (∫_0_^*T*^ *u*_2_^2^(*t*)*dt* = 55), and *W*_3_ · (∫_0_^*T*^ *u*_3_^2^(*t*)*dt* = 60)—is $0.6125 with a daily cost of $0.0001.

**Figure 4:**
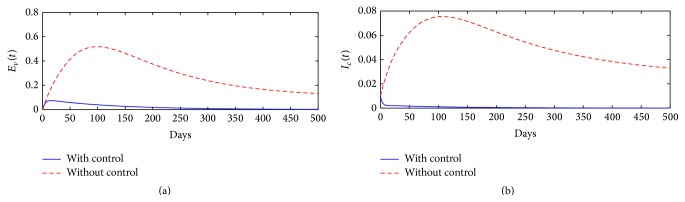
Time-series plots showing the impact of optimal intervention strategies on controlling FMD prevalence in the community, over a period of 500 days.

Figure 4 shows the long-term dynamics of FMD in epidemiological classes *E*_*v*_(*t*) and *I*_*c*_(*t*). We note that, in the presence of controls, the epidemiological class *E*_*v*_(*t*) dies out after 350 days of implementing these methods, while *I*_*c*_(*t*) dies out after 200 days. The overall conclusion of the simulations is that all FMD cases can be eliminated from the community after 350 days of implementing the aforementioned control methods.

**Figure 5:**
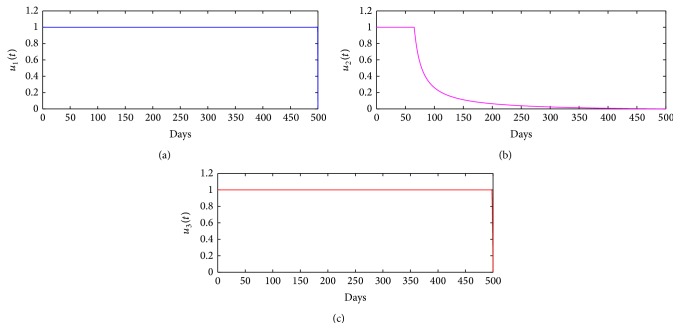
Control profiles for control function: (a) *u*_1_(*t*), (b) *u*_2_(*t*), and (c) *u*_3_(*t*), over a period of 500 days.

Figure 5 displays the time-dependent controls, *u*_1_(*t*), *u*_2_(*t*), and *u*_3_(*t*), over a period of 500 days. The simulations suggest that vaccination control, *u*_1_, and culling of infectious nonsymptomatic animals control, *u*_3_, must be maximized for the entire period of 500 days during which the interventions lasts, while detection and culling of infectious symptomatic animals control, *u*_2_, should stay at its maximum intensity, for approximately 65 days, and then can be progressively reduced. The total relative cost for the three control strategies over a long time horizon, 500 days, is computed as follows: *W*_1_ · (∫_0_^*T*^ *u*_1_^2^(*t*)*dt* = 500), *W*_2_ · (∫_0_^*T*^ *u*_2_^2^(*t*)*dt* = 282.5), and *W*_3_ · (∫_0_^*T*^ *u*_3_^2^(*t*)*dt* = 500) to get $0.045 with a daily cost of $0.00009. The costs for the short and long time horizons seem “roughly” comparable; in fact the daily costs are approximately the same.

*Efficacy of Optimal Intervention Strategy*. The efficacy of an intervention strategy on curbing new infections reflects the strength of the strategy to effectively control the epidemic. In this section, we explore the effectiveness of the aforementioned optimal intervention methods on reducing cumulative infectious FMD cases. We define the efficacy function *E*_*f*_(*t*) by

(11) $$\begin{array}{c} E_{f}\left(\;t\;\right)=1-\left[\;\frac{I^{*}\left(t\right)+I_{c}^{*}\left(t\right)}{I\left(0\right)+I_{c}\left(0\right)}\;\right], \end{array}$$

where *I* ^*∗*^(*t*) and *I*_*c*_^*∗*^(*t*) denote the optimal solutions associated with the optimal control of the corresponding variable and *I*(0) and *I*_*c*_(0) denote the corresponding initial condition. The function (11) measures the proportional decrease in the number of FMD infectious animals imposed by the intervention with controls, *u*_1_, *u*_2_, *u*_3_, by comparing the number of FMD infectious animals at time *t* with the initial conditions for which there are no controls implemented; that is, *u*_1_ = *u*_2_ = *u*_3_ = 0. By construction, *E*(*t*)∈[0,1] for all time *t*. Thus the upper bound of *E*(*t*) is one.

**Figure 6:**
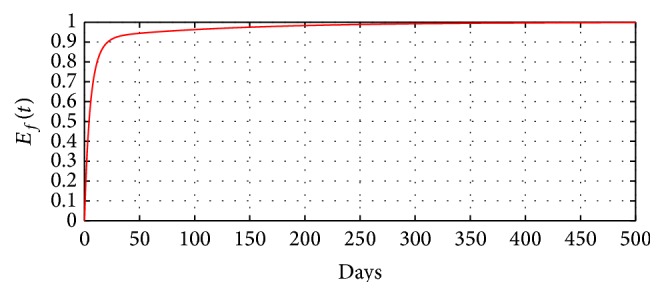
Time-series plot demonstrating the efficacy of optimal intervention strategies over a period of 500 days.

Figure 6 illustrates the effectiveness of optimal intervention methods aimed at curtailing FMD in the community over a period of 500 days. We note that, after 50 days of implementing the strategies, the efficiency level will be above 90% and will reach the 100% mark after 300 days. This demonstrates that optimal intervention strategies can be effective in reducing or eliminating new FMD infections in the community.

## 3. Concluding Remarks

We have developed a dynamic model for foot-and-mouth disease (FMD). In this work, our research is focused on proposing the “optimal prevention and control strategy of FMD” from mathematical modeling. We have introduced three control mechanisms representing pre- and postexposure vaccination, culling of symptomatic animals, and culling of infectious nonsymptomatic animals into our model. The various strategies associated with these three controls have been investigated. Numerical simulations demonstrate that vaccination and case finding of infectious nonsymptomatic animals are the most effective controls. Hence, for effective control of FMD during an outbreak, these two controls should be maximized for the entire period.

If all three controls are used, then the number of latently infected (not vaccinated) *E*(*t*) and symptomatic *I*(*t*) will be almost zero in a period of 40 days. However, the number of animals in epidemiological classes *E*_*v*_(*t*) and *I*_*c*_(*t*) will be negligible in a period of 350 and 250 days, respectively. This shows that the optimal control strategy of FMD elimination can be effective in controlling the disease during an outbreak. Further, our analysis demonstrated that control, *u*_2_, (culling of infectious symptomatic animals) can be sustainable for a 100-day strategy; thereafter the control can be dropped.

Our model has several limitations, which should be acknowledged. We have assumed that infections can be transmitted through contact between an infectious and a susceptible animal, although airborne foot-and-mouth disease virus transmission has been documented [4]. Incorporating this aspect may bring a new dimension to our results. In practice, the movement of animals can be influenced by a number of factors such as seasonal variations. The current paper did not include such factors, though these might be as well worthwhile to model and analyze mathematically. Further, we assumed mass action incidence, although some researchers believe that there is little evidence that in ecology any form of contact among animals or individuals abides closely to this law [21].

## Figures and Tables

**Table 1:** Model parameters and their interpretations.

Description	Symbol	Units	Baseline value	Ref.
Vaccine efficacy	*θ*	—	0.5	[2]
Modification factor	*ϵ*	—	0.4	Estimate
FMD transmission rate	*β*	Day^−1^	0.6	[3]
Non-FMD related exit rate	*μ*	Day^−1^	0.001	[15]
Latency period for animals in class *E*	*γ*	Day^−1^	0.26	[11]
Latency period for animals in class *E*_*v*_	*ω*	Day^−1^	0.07	[11]
Fraction of exposed animals vaccinated	*f*	—	0.65	Estimate
FMD-related death for animals in class *I*_*c*_	*δ*	Day^−1^	0.0008	[5]
FMD-related permanent exit from class *I*	*d*	Day^−1^	0.143	[3]
Detection and culling of infectious FMD carriers	*ξ*	Day^−1^	0.25 (0–0.25)	[3]
Rate of vaccination for the susceptible population	*α*	Day^−1^	0.25	[3]
Proportion of infectious animals detected and culled	*σ*	—	0.85	Estimate
Proportion of infected animals that become carriers	*κ*	—	0.45 (0.15–0.5)	[5]

**Table 2:** Sensitivity indices of *ℛ*_*A*_ to parameters for the FMD model (2), evaluated at the baseline parameter values given in Table 1.

Parameter	Sensitivity index
*β*	+1
*θ*	−0.99
*ξ*	−0.99
*ϵ*	−0.66
*ω*	+0.014
*μ*	−0.010
*α*	−0.0079
*f*	−0.0075
*σ*	−0.0070
*d*	−0.0052
*κ*	−0.0033
*δ*	−0.0031
*γ*	+0.00005

## Appendices

### A. Stability Analysis of Model Steady States

#### A.1. Global Stability of the Disease-Free Equilibrium

Following Castillo-Chavez et al. (2002) [22], system (2) is presented in the form

(A.1) X′t=FX,Y,Y′t=GX,Y,GX,0=0,

where *X* = (*S*, *V*) and *Y* = (*E*, *E*_*v*_, *I*, *I*_*c*_). Here *X* ∈ *ℝ*_+_^2^ denotes the uninfected population and *Y* ∈ *ℝ*_+_^4^ denotes the infected population. The infection-free equilibrium point is now denoted by *ℰ* ^0^ = (*X*_0_, 0) where *X*_0_ = [*μ*/(*α* + *μ*), *α*/(*α* + *μ*)]. We have to prove that following the two conditions

(H1) For *X*′(*t*) = *F*(*X*, 0), *X* is globally asymptotically stable,
(H2) $\hat{G}(X,Y)=UY-G(X,Y),\;\;\hat{G}(X,Y)\geq 0$ for (*X*, *Y*) ∈ *Ω*,

are satisfied, where *Ω* is a positively invariant attracting domain. Consider

(A.2) FX,0=μ−μ+αSαS−μV,U=−μ+γ0βμα+μβ1−ϵμα+μfγ−μ+ωβ1−θαα+μβ1−ϵ1−θαα+μ1−f1−κγ0−μ+σd01−fκγω1−σd−μ+ξ+δ.

Thus,

(A.3) G^X,Y=βI+1−ϵIcμα+μ−SβI+1−ϵIcαα+μ−V00.

Since *μ*/(*α* + *μ*) ≤ *S* and *α*/(*α* + *μ*) ≤ *V* at *ℰ* ^0^, it follows that $\hat{G}(X,Z)\geq 0$. Hence *ℰ* ^0^ is globally asymptotically stable whenever *ℛ*_*A*_ ≤ 1.

#### A.2. Endemic Equilibrium and Its Local Stability Analysis

Solving system (2) in terms of *λ* ^*∗*^ = *β*[*I* ^*∗*^ + (1 − *ϵ*)*I*_*c*_^*∗*^] yields

(A.4) E∗=S∗=μλ∗+α+μ,V∗=αμ1−θλ+μλ+α+μ,E∗=λ∗μμ+γλ∗+α+μ,I∗=1−κ1−fγλ∗μμ+σdμ+γλ∗+α+μ,Ev∗=1−θλ∗αμμ+ω1−θλ∗+μλ∗+α+μ+fγλ∗μμ+γλ∗+α+μ,Ic∗=1−θλ∗αμωμ+ξ+δμ+ω1−θλ∗+μλ∗+α+μ+fγμωλ∗+1−fκγμλ∗μ+ξ+δμ+γλ∗+α+μ+1−σd1−κ1−fγμλ∗μ+ξ+δμ+σdμ+γλ∗+α+μ.

Substituting *I* ^*∗*^ and *I*_*c*_^*∗*^ into *λ* ^*∗*^ = *β*[*I* ^*∗*^ + (1 − *ϵ*)*I*_*c*_^*∗*^], one gets

(A.5) Fλ∗=βμλ∗+α+μA1+1−κ1−fγ+1−κ1−f1−σdμ+ξ+δ,

where

(A.6) A1=1−ϵμ+σdαω1−θμ+γ1−θλ∗+μμ+ωμ+ξ+δ+1−ϵfγω+μ+ω1−fκγμ+ωμ+ξ+δ,

such that there exists an endemic equilibrium for model (2) if and only if there is a positive solution to *F*(*λ* ^*∗*^) = 1. Because

(A.7) F0=RA,limλ∗→∞⁡Fλ∗=0,

there exists an endemic equilibrium if *ℛ*_*A*_ > 1. Further, global stability of *ℰ* ^0^ implies that *ℰ* ^*∗*^ is a unique endemic equilibrium point.

We now use the Centre Manifold Theory [23] to investigate the local stability of *ℰ* ^*∗*^ whenever *ℛ*_*A*_ > 1 but close to one.

The Jacobian matrix of system (2) evaluated about *ℰ* ^0^ is

(A.8)

Let *β* = *β* ^*∗*^ be the bifurcation parameter. From (A.8), we can deduce that the right eigenvectors are

(A.9) w1=−β∗μw5+1−ϵw6α+μ2,w2=−β∗αw5+1−ϵw6μμ+α1−θ+μα+μ,w3=β∗μw5+1−ϵw6μ+αμ+γ,w4=β∗w5+1−ϵw6α+μμ+ωα1−θ+fγμμ+γ,w5=1−f1−κγμ+σdw3,w6>0.

Further, the left eigenvector of *J* associated with the zero eigenvalue at *β* = *β* ^*∗*^ is given by *v* = [*v*_1_, *v*_2_, *v*_3_, *v*_4_, *v*_5_, *v*_6_]^*T*^, where

(A.10) v1=v2=0,v3=γfv4+1−f1−κv5+κv6μ+γ,v4=ωμ+ωv6,v5=β∗μv3+α1−θv5μ+σdμ+α+1−σdμ+σdv6,v6>0.

It can easily be deduced that the bifurcation coefficients *a* and *b* are given by

(A.11) a=−2μv3+1−θα+μ1−θ+μμv4·β∗w5+1−ϵw6α+μ2<0,b=μv3+αv4w5+1−ϵw6α+μ>0.

Since *a* < 0 and *b* > 0, then Theorem 2 holds.

### B. Proof of Theorem 1

Proof The Hamiltonian *H* associated with the problem (7)–(10) is given by

(B.1) H=S+E+I+Ic+W12u12+W22u22+W32u32+λ1μ−βI+1−ϵIcS−μ+u1αS+λ2u1αS−β1−θI+1−ϵIcV−μV+λ3βI+1−ϵIcS−μ+γE+λ4β1−θI+1−ϵIcV+u1γE−μ+ωEv+λ51−u11−κγE−μ+u2dI+λ6ωEv+1−u1κγE+1−u2dI−μ+ξu3+δIc,

where *λ*_*i*_(*t*), *i* = 1,2, 3,4, 5,6, denotes the adjoint functions associated with the states *S*, *E*, *E*_*v*_, *I*, *I*_*c*_, respectively. Note that, in *H*, each adjoint function multiplies the right-hand side of the differential equation of its corresponding state function. The first term in *H* comes from the integrand of the objective functional. Given an optimal control triple *u*_1_^*∗*^, *u*_2_^*∗*^, *u*_3_^*∗*^ and corresponding states (*S* ^*∗*^, *V* ^*∗*^, *E* ^*∗*^, *E*_*v*_^*∗*^, *I* ^*∗*^, *I*_*c*_^*∗*^), there exist adjoint functions satisfying

(B.2) λ1∗˙t=−1+μλ1∗+αu1∗λ1∗−λ2∗+βI∗+1−ϵIc∗λ1∗−λ3∗,λ2∗˙t=μλ2∗+β1−θI∗+1−ϵIc∗λ2∗−λ4∗,λ3∗˙t=−1+μλ3∗+γλ3∗−u1∗λ4∗−1−κ1−u1∗λ5∗−1−u1∗κλ6∗,λ4∗˙t=μλ4∗+ωλ4∗−λ6∗,λ5∗˙t=−1+μλ5∗+du2∗λ5∗−1−u2∗λ6∗+βS∗λ1∗−λ3∗+βV∗1−θλ2∗−λ4∗,λ6∗˙t=−1+μ+δ+ξu3λ6∗+βS∗1−ϵIc∗λ1∗−λ3∗+βV∗1−ϵ1−θλ2∗−λ4∗,

with transversality conditions *λ*_*j*_(*T*) = 0, for *j* = 1,2,…, 6. Furthermore, the optimal controls are characterised by

(B.3) u1∗t=min⁡max⁡0,λ1∗−λ2∗αS∗W1+λ5∗−λ4∗+κλ6∗−λ5∗γE∗W1,1,u2∗t=min⁡max⁡0,dλ5∗+λ6∗dI∗W2,1,u3∗t=min⁡max⁡0,λ6∗ξIc∗W3,1.
